# Prognostic Role of Pretreatment Prognostic Nutritional Index in Advanced Lung Cancer Patients Receiving First-Line Immunotherapy: A Meta-Analysis

**DOI:** 10.7759/cureus.52720

**Published:** 2024-01-22

**Authors:** Junrong Yang, Hui Li, Liangqin Li, Jing Lv

**Affiliations:** 1 Department of Thoracic Surgery, People’s Hospital of Deyang City, Deyang, CHN

**Keywords:** meta-analysis, prognosis, first-line immunotherapy, advanced lung cancer, prognostic nutritional index

## Abstract

The aim of this study was to further explore the association between pretreatment prognostic nutritional index (PNI) and survival among advanced lung cancer patients who received the first-line immunotherapy based on current relevant studies. Several databases were searched up to July 17, 2023. Progression-free survival (PFS) and overall survival (OS) were primary outcomes and the hazard ratios (HRs) with 95% confidence intervals (CIs) were combined. Subgroup analysis based on the pathological type [non-small cell lung cancer (NSCLC) vs small cell lung cancer (SCLC)] and combination of other therapies (yes vs no) were performed. Ten studies with 1291 patients were included eventually. The pooled results demonstrated that higher pretreatment PNI was significantly related to improved PFS (HR=0.62, 95% CI: 0.48-0.80, P＜0.001) and OS (HR=0.52, 95% CI: 0.37-0.73, P＜0.001). Subgroup analysis revealed that the predictive role of pretreatment PNI for PFS (HR=0.61, 95% CI: 0.45-0.81, P=0.001) and OS (HR=0.52, 95% CI: 0.35-0.77, P=0.001) was only observed among NSCLC patients and the combination of other therapies did not cause an impact on the prognostic role of PNI in lung cancer. Pretreatment PNI was significantly associated with prognosis in advanced NSCLC receiving first-line immunotherapy and patients with a lower pretreatment PNI had poorer survival.

## Introduction and background

Lung cancer remains the most common malignancy and leading cause of tumor-related deaths over the world [1,2]. It consists of non-small cell lung cancer (NSCLC) and small cell lung cancer (SCLC). Despite the great advances in early screening and surgical technologies, a significant proportion of lung cancer patients are diagnosed with advanced stage or relapse after initial treatment, which causes the high mortality rate of lung cancer [1,2]. For advanced-stage lung cancer, immunotherapy based on immune checkpoint inhibitors (ICIs) has been one of the important therapies in recent years and greatly contributes to the improvement of prognosis in patients with negative driver mutations [3,4].

Although it has been reported that ICIs including the programmed death-ligand 1 (PD-L1) and programmed death-1 (PD-1) and also cytotoxic T-lymphocyte-associated protein 4 (CTLA-4) inhibitors could significantly improve the survival among advanced lung cancer patients overall, there is still a proportion of lung cancer patients who do not respond well to immunotherapy [5,6]. Thus, it is essential to accurately clarify the potential beneficiaries of immunotherapy. Up to now, the expression of PD-L1 and tumor mutational burden (TMB) are main indicators predicting the therapeutic effect of ICIs in clinics. However, they do not always show good predictive value. In detail, lung cancer patients with low PD-L1 expression might benefit from the immunotherapy and patients with high PD-L1 expression may also experience the failure of immunotherapy [7]. Besides, the clinical application of these two indicators might be limited due to the high cost of detection and heterogeneity of tumor specimens. Therefore, it is urgently needed to identify more simple, inexpensive and readily available biomarkers that could predict the efficacy of ICIs.

Prognostic nutritional index (PNI) is a novel index based on the serum albumin level and peripheral absolute lymphocyte count. Initially, it was developed to assess the preoperative nutritional status and postoperative complication risk [8]. A number of studies have indicated that it is a valuable index predicting the prognosis of lung cancer patients including patients at advanced stage [9,10]. It is suggested that anti-tumor treatments might have an impact on the values of PNI. Thus, it is controversial to identify the prognostic value of pre-immunotherapy PNI in lung cancer patients receiving second-line or third-line immunotherapy. However, the association of pretreatment PNI with therapeutic efficacy of first-line immunotherapy among advanced-stage lung cancer remains unclear now.

Therefore, this meta-analysis aimed to further clarify the predictive role of pretreatment PNI for prognosis in advanced lung cancer patients receiving first-line immunotherapy.

## Review

Materials and methods

This meta-analysis was conducted according to the Preferred Reporting Items for Systematic Review and Meta-Analyses (PRISMA) 2020 [11].

Literature Search

The Web of Science, EMBASE, PubMed, Cochrane Library and CNKI databases were searched from their inception to July 17, 2023 and the following terms were used: PD-1, PD-L1, CTLA-4, ICIs, immune checkpoint inhibitor, lung, pulmonary, tumor, cancer, carcinoma, neoplasm, prognostic nutritional index, PNI, survival, prognosis and prognostic. Detailed search strategy was as follows: (PD-1 OR PD-L1 OR CTLA-4 OR ICIs OR immune checkpoint inhibitor) AND (lung OR pulmonary) AND (tumor OR cancer OR carcinoma OR neoplasm) AND (prognostic nutritional index OR PNI) AND (survival OR prognosis OR prognostic). Meanwhile, Medical Subject Heading (MeSH) terms and free texts were applied and all references in the included studies were reviewed.

Inclusion Criteria

Studies that met the following criteria were included: 1) patients were pathologically diagnosed with primary lung cancer and with advanced stage; 2) the immunotherapy was received as first-line anti-tumor treatment; 3) the pre-immunotherapy PNI was defined as 10 × albumin (g/dL) + 0.005 × absolute lymphocyte count (/µL); 4) the association of pre-immunotherapy PNI with progression-free survival (PFS) or (and) overall survival (OS) was explored; 5) hazard ratios (HRs) with 95% confidence intervals (CIs) were reported directly; 6) full texts were available.

Exclusion Criteria

Studies that met the following criteria were excluded: 1) insufficient or duplicated data; 2) low-quality studies with a Newcastle-Ottawa Scale (NOS) score of 5 or lower; 3) letters, editorials, case reports, reviews or animal trials.

Data Collection

We collected the following information from each included study: the first author, publication year, country, sample size, pathological type (NSCLC or SCLC), combination therapy such as the chemotherapy and targeted therapy, ICI drugs such as pembrolizumab, nivolumab, bevacizumab, durvalumab, sintilimab, tislelizumab and atezolizumab, cutoff value of PNI, endpoints including the PFS and OS, HR and 95% CI.

Methodological Quality Assessment

All included studies were retrospective. Thus, the NOS tool was applied to evaluate the methodological quality. As mentioned above, only studies with a NOS score ≥ 6 were included.

The literature search, selection, information collection and quality assessment were performed by two authors independently.

Statistical Analysis

All analyses were performed by Stata version 15.0 (StataCorp., College Station, TX, USA). The heterogeneity between studies was assessed by using I^2^ statistics and the Q test. If significant heterogeneity was detected representing as I^2^ > 50% and/or P < 0.1, the random effects model was applied; or the fixed effects model was applied [12]. HRs and 95% CIs were combined to evaluate the relationship between pretreatment PNI and survival. Subgroup analysis based on pathological type (NSCLC vs SCLC) and combination of other therapies (yes vs no) was further performed. Sensitivity analysis was conducted to detect the sources of heterogeneity and assess the stability of the overall results. Besides, Begg’s funnel plot and Egger’s test were conducted to detect publication bias, and significant publication bias was defined as P < 0.05 [13,14].

Results

Literature Search and Selection Process

**Figure 1:**
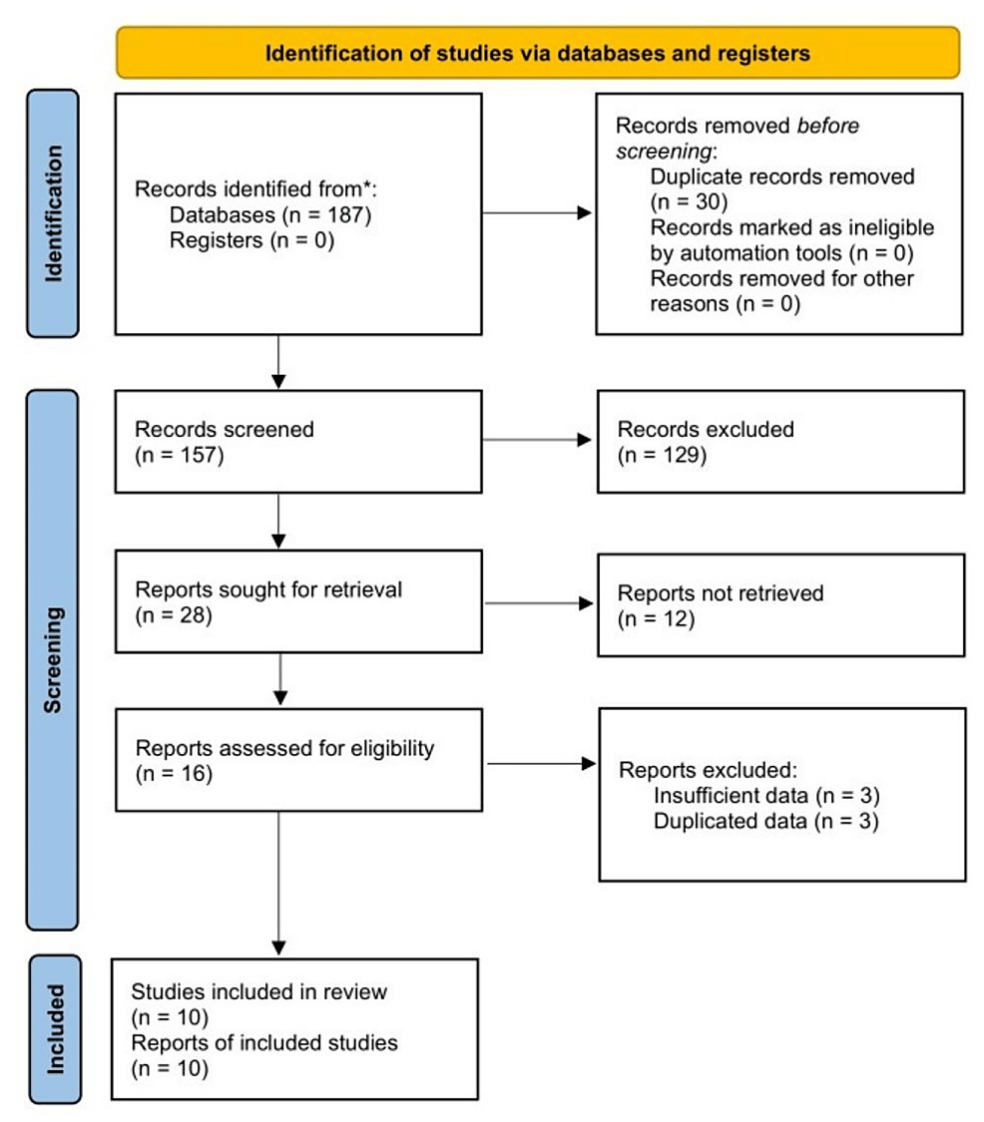
Preferred Reporting Items for Systematic Review and Meta-Analyses (PRISMA) flow diagram of this meta-analysis.

One hundred and eighty-seven records were identified from the five databases. After reviewing the titles, abstracts and full texts, 10 studies were included in this meta-analysis [15-24]. Detailed selection process is shown in Figure 1.

Basic Characteristics of Included Studies

All included studies were retrospective and 1291 cases were enrolled with the sample size ranging from 34 to 237. Most studies were from China or Japan and only two studies focused on SCLC. Besides, most patients received a combination of chemotherapy. Notably, in the studies by Yang et al. [17] and Oku et al. [22], two subgroups of individuals receiving single immunotherapy and combined therapy were analyzed separately. Therefore, in our meta-analysis, the two subgroups in their studies were also analyzed separately. Detailed information is presented in Table 1.

**Table 1:** Basic characteristics of included studies. NSCLC: non-small cell lung cancer; SCLC: small cell lung cancer; LC: lung cancer; PD-1: programmed death 1; PNI: prognostic nutritional index; PFS: progression-free survival; OS: overall survival; NOS: Newcastle-Ottawa Scale.

Author	Year	Country	Sample size	Pathological type	Combined treatment	Immunotherapy drugs	Cutoff value of PNI	Endpoint	NOS
Ogura [15]	2020	Japan	34	NSCLC	Chemotherapy	Atezolizumab, Bevacizumab and Pembrolizumab	40	PFS, OS	6
Qi [16]	2021	China	53	SCLC	Chemotherapy	Atezolizumab	48	OS	6
Yang [17]	2021	China	45	NSCLC	No/Chemotherapy/Targeted therapy/Others	Pembrolizumab,Nivolumab, Sintilimab, Tislelizumab and Atezolizumab	52.8	PFS	7
Fang [18]	2022	China	223	NSCLC	Chemotherapy	PD-1 inhibitor	50.5	PFS	7
Stares [19]	2022	UK	219	NSCLC	No	Pembrolizumab	45	PFS, OS	8
Shijubou [20]	2022	Japan	38	NSCLC	No	Pembrolizumab	40	PFS	6
Tanaka [21]	2022	Japan	237	NSCLC	Chemotherapy	Pembrolizumab, Atezolizumab and Bevacizumab	40.35	PFS, OS	8
Oku [22]	2023	Japan	91	NSCLC	No/Chemotherapy	Pembrolizumab and Atezolizumab	42.17	PFS, OS	8
Takeda [23]	2023	Japan	155	SCLC	Chemotherapy	Atezolizumab, Durvalumab	40	PFS, OS	7
Han [24]	2023	China	69	NSCLC	Chemotherapy	Pembrolizumab and Nivolumab	41.75	PFS	6

The Association of Pretreatment PNI With PFS in Advanced Lung Cancer Receiving the First-Line Immunotherapy

Nine studies clarified the predictive role of pretreatment PNI for PFS [15,17-24]. Our pooled results manifested that a lower pretreatment PNI predicted worse PFS (HR=0.62, 95% CI: 0.48-0.80, P＜0.001; I^2^=50.9%, P=0.026).

Then, subgroup analyses based on the pathological type and combination of other therapies were further conducted. The significant relationship between pretreatment PNI and PFS was only observed among NSCLC patients (NSCLC: HR=0.61, 95% CI: 0.45-0.81, P=0.001; SCLC: HR=0.70, 95% CI: 0.46-1.07, P=0.098). The combination of other therapies (mainly chemotherapy) did not affect the prognostic value of PNI in lung cancer (yes: HR=0.59, 95% CI: 0.43-0.83, P=0.002; no: HR=0.66, 95% CI: 0.42-1.04, P=0.074), although the association between PNI and PFS in patients receiving immune-monotherapy did not reach statistical difference (Table 2).

**Table 2:** Results of meta-analysis. NSCLC: non-small cell lung cancer; SCLC: small cell lung cancer.

	No. of studies	Hazard ratio	95% confidence interval	P value	I^2^(%)	P value for heterogeneity
Progression-free survival	9	0.62	0.48-0.80	＜0.001	50.9	0.026
Pathological type						
NSCLC	8	0.61	0.45-0.81	0.001	55.6	0.016
SCLC	1	0.70	0.46-1.07	0.098	-	-
Combination therapy						
Yes	6	0.59	0.43-0.83	0.002	59.6	0.021
No	4	0.66	0.42-1.04	0.074	41.2	0.164
Overall survival	7	0.52	0.37-0.73	＜0.001	64.5	0.006
Pathological type						
NSCLC	5	0.52	0.35-0.77	0.001	67.0	0.010
SCLC	2	0.57	0.19-1.72	0.321	71.5	0.061
Combination therapy						
Yes	6	0.56	0.36-0.87	0.011	68.5	0.007
No	2	0.43	0.30-0.61	＜0.001	9.5	0.293

The Association of Pretreatment PNI With OS in Advanced Lung Cancer Receiving the First-Line Immunotherapy

Seven studies explored the relationship of pretreatment PNI with OS [15,16,18,19,21-23]. The pooled results showed that PNI was significantly associated with OS (HR=0.52, 95% CI: 0.37-0.73, P＜0.001; I^2^=64.5%, P=0.006).

Similarly, the predictive role of pretreatment PNI for OS was only detected among NSCLC patients (NSCLC: HR=0.52, 95% CI: 0.35-0.77, P=0.001; SCLC: HR=0.57, 95% CI: 0.19-1.72, P=0.321). Furthermore, the combination of chemotherapy did not show an impact on the prognostic role of PNI (yes: HR=0.56, 95% CI: 0.36-0.87, P=0.011; no: HR=0.43, 95% CI: 0.30-0.61, P＜0.001) (Table 2).

Sensitivity Analysis and Publication Bias

**Figure 2:**
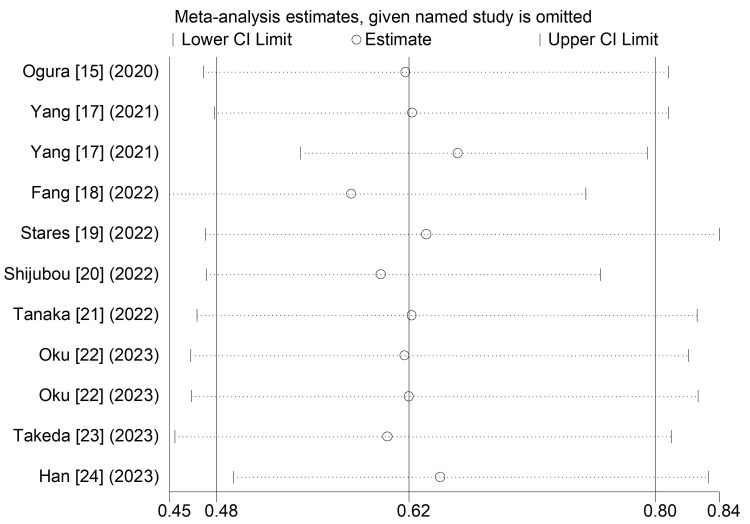
Sensitivity analysis for the association between pretreatment prognostic nutritional index and progression-free survival in advanced lung cancer receiving the first-line immunotherapy.

Sensitivity analysis for the PFS involving nine included studies (Figure 2) was conducted, which indicated that our results were stable and reliable.

**Figure 3:**
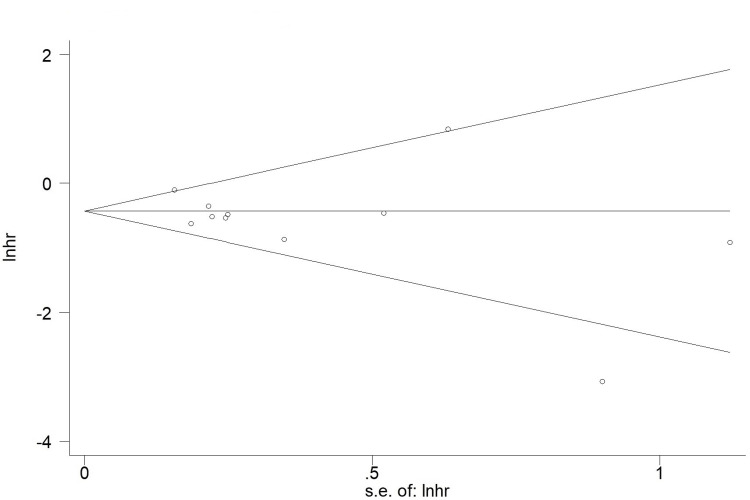
Begg’s funnel plots for the association between pretreatment prognostic nutritional index and progression-free survival in advanced lung cancer receiving the first-line immunotherapy. hr: hazard ratio; se: standard error

According to the Begg’s funnel plots for PFS involving nine included studies (Figure 3) and P values of Egger’s test (P=0.274), no obvious publication bias was observed in this meta-analysis.

Discussion

PNI is calculated based on the serum albumin concentration and peripheral lymphocyte count, which could well reflect the nutritional and immune status of patients. This study was the first meta-analysis that clarified the prognostic role of pretreatment PNI in advanced-stage lung cancer patients who received the first-line immunotherapy and our results demonstrated that a lower pretreatment PNI indicated worse survival among NSCLC patients. Besides, PNI could well predict the prognosis for patients with or without the combination of other therapies, mainly chemotherapy. Thus, PNI could serve as a valuable and reliable indicator for the screening of potential beneficiaries of immunotherapy.

Actually, the prognostic value of PNI in lung cancer has also been identified by several meta-analyses. In 2018, Wang et al. included 21 studies and demonstrated that low PNI was related to shorter OS (HR=1.59, 95% CI: 1.28-1.96, P=0.001), PFS (HR=1.52, 95% CI: 1.26-1.83, P=0.002) and disease-free survival (DFS)/relapse-free survival (RFS) (HR=1.74, 95% CI: 1.08-2.80, P=0.017) [25]. Besides, Hu et al. included 15 studies and showed that a low pretreatment PNI was significantly associated with adverse OS (HR=1.61, 95% CI: 1.44-1.81, P＜0.001), PFS (HR=1.52, 95% CI: 1.26-1.83, P=0.002) and DFS/RFS (HR=2.27, 95% CI: 1.40-3.69, P＜0.01) [26]. Then another meta-analysis by Zhang et al. indicated that PNI was also related to the PFS (HR=1.31) and OS (HR=1.21) among lung cancer patients receiving chemotherapy [27]. However, advanced lung cancer patients receiving first-line immunotherapy are a relatively special population and the relationship between pretreatment PNI and survival of this group of patients remains unclear before this study.

In this meta-analysis we first demonstrated that pre-immunotherapy PNI was a valuable predictor for PFS and OS in advanced-stage NSCLC patients who received first-line immunotherapy. Notably, although no significant association between pretreatment PNI and survival was observed among SCLC patients, two included studies focusing on SCLC both indicated a certain trend [16,23]. Besides, only 208 participants were enrolled in these two studies. Furthermore, Jiang et al. included nine studies with 4164 SCLC patients and verified that lower PNI was significantly related to worse OS in SCLC (HR=1.43, 95% CI: 1.24-1.64, P＜0.001) among SCLC patients [28]. Therefore, we deem that it is still needed to further explore the predictive role of pretreatment PNI for the prognosis in extensive-stage SCLC receiving first-line immunotherapy combined with chemotherapy.

Regarding the clinical role of PNI in advanced-stage lung cancer, we deem that some investigations should be further performed in future relevant studies. For example, it is useful to clarify the impact of intervention in the PNI on the efficacy of ICIs. In other words, increasing the PNI value during the therapy may improve the therapeutic effect of ICIs in lung cancer, which should be further determined. Besides, a combination of other systemic inflammation and nutritional indicators such as the systemic immune-inflammation index (SII) and lung immune prognostic index (LIPI) may show higher prognostic value in advanced lung cancer receiving first-line immunotherapy. Furthermore, the association between the change of PNI during the immunotherapy and prognosis should also be investigated.

There are some limitations that exist in our meta-analysis. First, the overall sample size is relatively small and fewer than 100 participants were enrolled in some included studies. Secondly, all included studies are retrospective. Thirdly, some confounding factors exist in this study such as the age, drugs of ICIs and cutoff values of PNI and we are unable to conduct more subgroup analyses based on these parameters. Fourthly, due to the lack of relevant data, we failed to explore the association between PNI and short-term outcomes in advanced lung cancer patients receiving the first-line immunotherapy.

## Conclusions

Pretreatment PNI was significantly associated with prognosis in advanced NSCLC receiving first-line immunotherapy and patients with a lower pretreatment PNI had poorer survival. More prospective high-quality studies are needed to further verify our findings due to the limitations exists in this meta-analysis.
